# Case Report: An innovative approach to coronary artery perforation in chronic total occlusion using autologous flaps

**DOI:** 10.3389/fcvm.2025.1594967

**Published:** 2025-05-27

**Authors:** Chen Genrui, Wang Huan, Chen Youhu, Li Chengxiang, Gao Haokao

**Affiliations:** Department of Cardiology, The First Affiliated Hospital of Air Force Military Medical University, Xi’an, China

**Keywords:** case report, chronic total occlusion (CTO), percutaneous coronary intervention (PCI), coronary artery perforation (CAP), the dissection and re-entry techniques (DARTs)

## Abstract

**Background:**

A percutaneous coronary intervention (PCI) for chronic total occlusion (CTO) carries a significant risk of coronary artery perforation (CAP). The typical treatment for proximal large CAP often requires the deployment of covered stents. However, this becomes impractical in situations where the antegrade wire has not successfully crossed the CTO lesion. In addition, emergency coronary artery bypass grafting for perforation after CTO-PCI is associated with a high in-hospital mortality rate.

**Case report:**

A patient presented with a left anterior descending artery (LAD) CTO involving the large first diagonal (D1) branch within the CTO segment. The D1 vessel was recanalized successfully using an antegrade approach. Before several attempts, the LAD CTO could n't be crossed, and an Ellis type Ⅲ perforation was visualized in the mid-LAD segment after bifurcation of the D1 vessel, which was created using a knuckled wire supported with a Corsair microcatheter. Subsequently, the perforation was effectively controlled by a tamponade balloon deployed from the D1 vessel to the proximal LAD. Before the retrograde wire crossed the LAD CTO, the dissection and re-entry techniques (DARTs) were used to recanalize the CTO lesion, intentionally creating subintimal dissection flaps. These autologous dissection flaps, together with drug-eluting stents instead of covered stents, successfully sealed the perforation.

**Conclusion:**

The innovative approach of using autologous dissection flaps created with DART in CTO-PCI to seal perforations is clinically feasible and effective.

## 1 Background

The occurrence of coronary artery perforation (CAP) is one of the most feared complications in percutaneous coronary intervention (PCI) for chronic total occlusion (CTO). It occurs in approximately 8% of CTO procedures and carries a high risk of periprocedural death (1). Although covered stents (CSs) can be applied to treat such perforations, an observed increase in the rates of restenosis and thrombosis has been noted during short-term follow-up (2). If the perforation occurs in the CTO body of a large main branch when wire access to the distal vessel has not been achieved, the deployment of covered stents becomes impractical. Once hemodynamic stability can no longer be maintained, emergency implantation of coronary stents from the side branch vessel to the main branch may be necessary. As a result, revascularization of the main branch may become more challenging in the future. Moreover, emergency surgery for the perforation entails a higher risk.

In this case report, we describe a patient who presented with an Ellis type III perforation during the antegrade approach for a left anterior descending artery (LAD) CTO-PCI. Subsequently, the CTO lesion was recanalized using the dissection and re-entry techniques (DARTs), with which dissection flaps were deliberately created. By utilizing these dissection flaps in combination with drug-eluting stents (DESs), the LAD perforation was successfully sealed.

## 2 Case report

**Figure 1:**
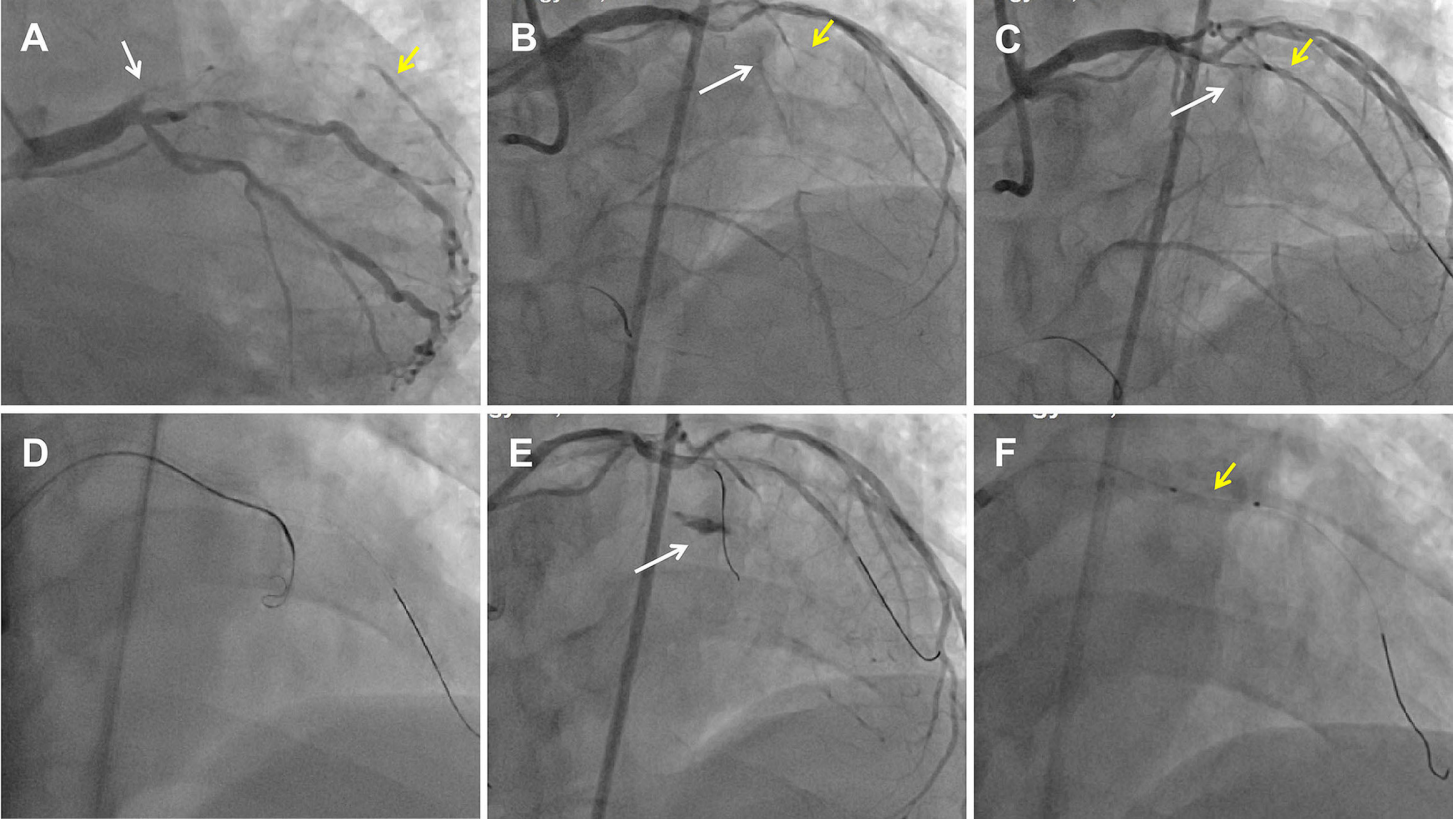
Perforation formation after an antegrade attempt during LAD CTO recanalization. **(A)** CAG showed a LAD CTO lesion with a stump (white arrow) and a D1 vessel (yellow arrow) filling by ipsilateral vessels. **(B)** Dual CAG showed middle LAD filling by collateral vessels from the RCA. The white arrow indicates the middle LAD, and the yellow arrow indicates the D1. **(C)** Pilot 200 wire into the distal true lumen of the D1 vessel. The white arrow indicates the mid-LAD segment, and the yellow arrow indicates the D1. **(D)** An antegrade Corsair catheter with a knuckled Pilot 200 wire was advanced into the supposed CTO body. **(E)** An Ellis type Ⅲ perforation occurred. The white arrow indicates contrast extravasation. **(F)** A proximal tamponade balloon (yellow arrow) from the D1 to the pro-LAD to control the perforation.

A 50-year-old man was referred to our hospital due to intermittent chest discomfort for 6 months, which had worsened over the previous 2 months despite receiving optimal medical therapy at maximum tolerance. The patient had a history of hypertension for 12 years, no family history of disease, and was on optimal medical therapy for coronary heart disease. After being admitted, the patient’s blood pressure (BP) was 125/80 mmHg, and his heart rate (HR) was 67 beats per minute (bpm). An electrocardiogram (ECG) showed ST elevation ≤0.05 mV in leads II, III, and aVF. Laboratory examination of cardiac enzymes was normal. Transthoracic echocardiography (TTE) showed a left ventricular ejection fraction (LVEF) of 56% with slight hypokinesis in the LV anterior and lateral walls. Diagnostic coronary angiography (CAG) revealed 80% stenosis in the left circumflex artery (LCX) and obtuse marginal artery 2 (OM2), along with total occlusion of the proximal LAD (Figure 1A). The patient and his family declined bypass graft surgery, and therefore, the primary strategy was to revascularize the LAD CTO lesion.

**Figure 2:**
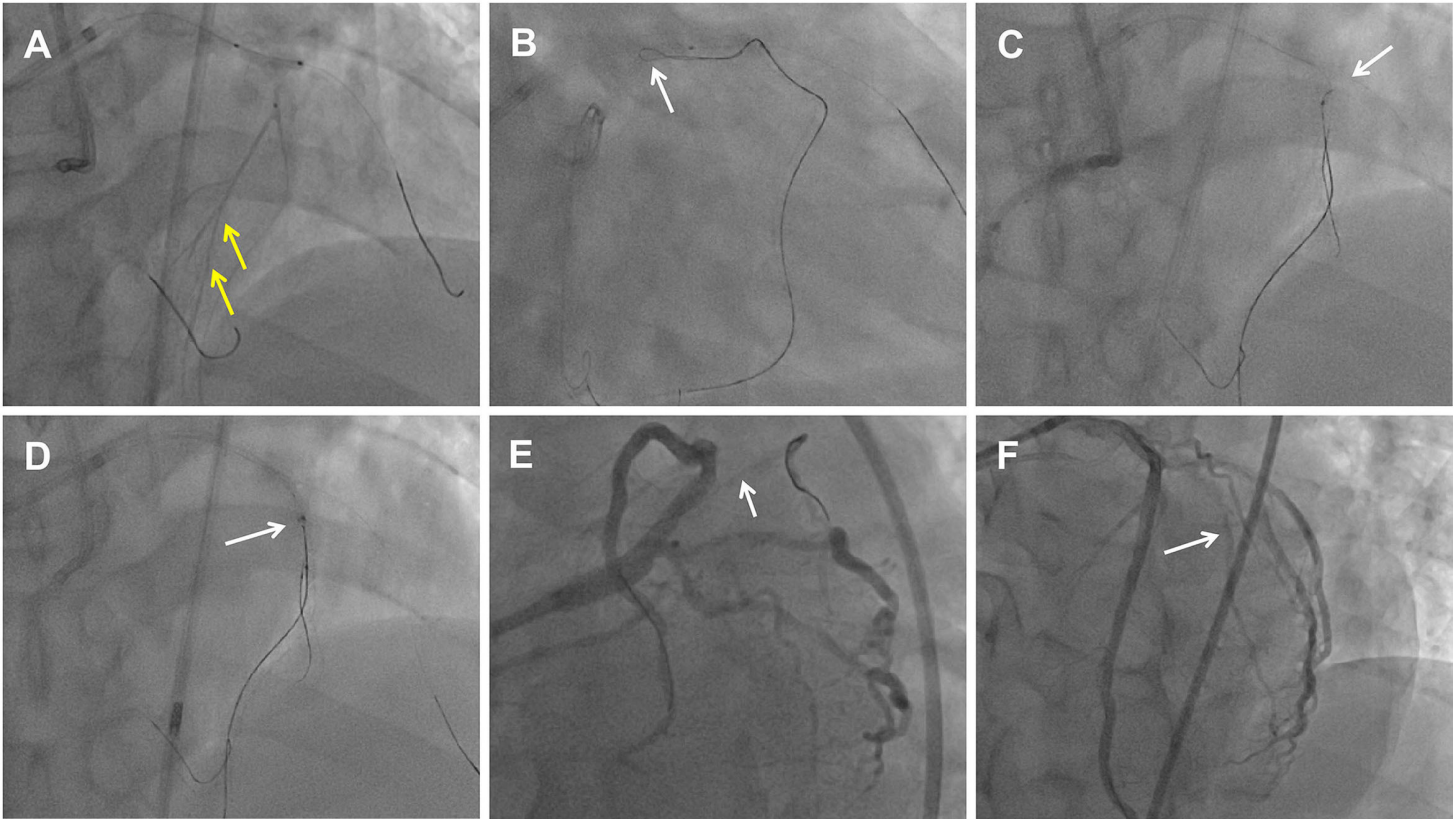
Dissection flaps created by the DART to seal the perforation with drug-eluting stent implantation. **(A)** Retrograde microcatheter tip injection showed collateral vessels connected to septal vessels (yellow arrow). **(B)** Retrograde knuckled wire (white arrow) into the proximal LAD CTO extra-plaque space. **(C)** Antegrade parallel wire into the vessel architecture and adjacent to the retrograde wire (white arrow). **(D)** Retrograde wire entered into the antegrade Guidezilla catheter (white arrow). **(E)** The ostium of the D1 vessel was crushed by the flaps which shifting after the LAD stent deployment. The white arrow indicates the D1 occlusion. **(F)** LAD and D1 vessel final angiogram results.

**Figure 3:**
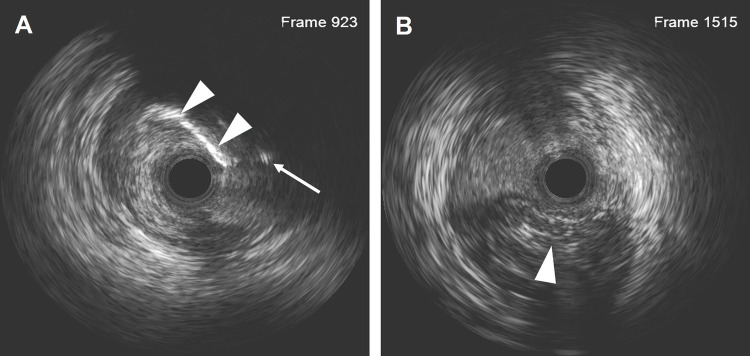
IVUS results after the LAD recanalization retracking sequences from the D1 vessel to the LAD and from the LAD distal true lumen. **(A)** The IVUS results showed that the retrograde wire entered the LAD vessel architecture through a calcification ring on the outer edge. The white arrow indicates the retrograde wire, and the white arrowhead indicates the calcification ring. **(B)** The IVUS results revealed that the wire was located in the subintimal space with a length of 18.2 mm. The arrowhead indicates the true lumen.

Dual angiography was performed and showed a proximal LAD CTO with a small stump, with ∼30 mm extending from the proximal to mid-LAD and several septal collaterals from the right coronary artery (RCA), and a good landing zone distal to the mid-LAD. Furthermore, it showed that the first large diagonal (D1) vessel was occluded within the LAD CTO lesion, and that two-thirds of its distal length was retrogradely filled via ipsilateral vessels, making it visualize as a larger vessel (Figure 1B, Supplementary Videos 1, 2). The primary antegrade approach was attempted with a Pilot 200 wire, which was successfully navigated into the distal true lumen of the D1 vessel (Figure 1C, Supplementary Video 3). After a 2.0 mm × 20 mm semi-compliant balloon dilation in the D1 vessel segment, dual CAG revealed that the LAD occlusion entry point was ambiguous. With the support of a dual lumen catheter (DLC), Gaia3 and Pilot 200 wires were used to explore the occlusion entrance, gaining access to the subintimal space. Subsequently, a knuckled Pilot 200 wire was tracked forward with a Corsair catheter, which appeared to be directed toward the septum rather than into the LAD course (Figure 1D, Supplementary Video 4), and intravascular ultrasound (IVUS) revealed that the entry point was incorrect. Next, several retrograde attempts were performed with softer wires such as SION/SION and Black/SUOH03 across the septal vessels, respectively, but these all failed. The following antegrade angiography revealed an Ellis type Ⅲ perforation (Figure 1E, Supplementary Video 5) located at the mid-LAD segment, which was likely caused by the knuckled Pilot 200 wire with the Corsair microcatheter tracking outside of the LAD architecture. Promptly, a 2.5 mm × 20 mm compliant balloon was used to tamponade from the D1 vessel to the proximal LAD and effectively controlled the pericardial bleeding (Figure 1F). In this situation, the retrograde approach was initiated again. The tip of the Sion wire, with a double-angled shape, crossed the RCA epicardial collateral vessel, which was connected to the septal vessels, as visualized by tip injection (Figure 2A, Supplementary Video 6). Stiffer wires, such as Pilot200/Gaia3, failed to penetrate the fibrous cap of the LAD CTO where it intersected with the D1 branch. Next, a knuckled Fielder XT-A wire was pushed forward into the LAD CTO body. Although reverse-controlled antegrade and retrograde tracking (r-CART) was performed, the UB3/Gaia3/CP12 stiffer wires all failed in the antegrade extension catheter (Figure 2B). Then, an antegrade parallel wire, with DLC support, was advanced as close as possible toward the retrograde wire (Figure 2C), and the extension catheter was subsequently delivered across the bifurcation, and successfully picked up the retrograde wire (Figure 2D). IVUS results, retracing from the D1 vessel to the LAD, confirmed that the retrograde wire entered the LAD vessel architecture through the outer edge of the calcification ring in the mid-LAD segment (Figure 3A), and from the LAD distal true lumen showed that the subintimal length was 18.2 mm (Figure 3B). To ensure adequate adhesion of the dissection flaps onto the perforation, multi-size balloon dilation for the D1 branch and the LAD, and 3.0 mm × 38 mm/3.5 mm × 38 mm DESs were deployed sequentially from the distal to the proximal LAD. IVUS was used to assess the optimal stent expansion and apposition. CAG showed that the perforation was completely sealed and there was thrombolysis in myocardial infarction (TIMI) grade III flow in the entire LAD (Figure 2E). Furthermore, the ostium of the D1 vessel was crushed, but good retrograde filling from the ipsilateral vessels was still observed (Figure 2F).

During the DART process, infusion and vasoactive drugs were administered to maintain the patient’s blood pressure at 100–120/60–80 mmHg and his HR at 80–100 bpm, without any malignant arrhythmias occurring. When the tamponade balloon for the perforation was intermittently deflated and his blood pressure decreased below 90/60 mmHg, high-dose vasoactive drugs were administered to maintain the mean arterial pressure at approximately 60 mmHg.

An X-ray image showed a significant amount of pericardial effusion. After performing pericardiocentesis and removing 200 ml of blood, there was a significant reduction in fluid accumulation. The activated clotting time (ACT) was 422 s, and 30 mg of protamine sulfate were administered to reverse the effects of heparin after the coronary gear was removed. The pericardial drainage was removed the next day. Three days later, the patient was discharged in a stable condition.

At the 6-month follow-up after the procedure, the patient underwent an examination of adenosine triphosphate (ATP) stress and rest-gated myocardial blood perfusion imaging, which showed that the movement of the LV anterior and interventricular walls was slightly to moderately decreased under ATP stress conditions, and both systolic and diastolic functions were also slightly decreased. At rest, the movement of each LV wall was close to normal, and both systolic and diastolic functions were normal. The LCX lesions remain untreated and are currently under close surveillance. At the 1-year clinical follow-up, the patient has remained free of angina symptoms.

## 3 Discussion

DARTs are a crucial way to improve the success rate in CTO-PCI. By following these techniques, dissection flaps can be created between the vessel lumen and the extra-plaque space; however, their shift may occlude the side branch (3, 4). Nevertheless, a subintimal shift to seal perforations has previously been described during retrograde RCA CTO-PCI (5, 6) with the use of r-CART, and during antegrade CTO-PCI (7) with the use of the subintimal tracking and re-entry technique (STAR). In this case, we applied DARTs to intentionally create efficient dissection flaps during LAD CTO retrograde recanalization, which were then utilized to seal a large vessel perforation.

The pivotal method to manage a CAP is to inflate a balloon proximal to the perforation, as we did in this case. Large vessel perforation during coronary intervention often requires the implantation of covered stents if the wire has crossed the CTO lesion and entered the distal vessel; otherwise, their use would be impractical. All currently available CTO crossing algorithms recommend the use of three strategies (IVUS-guided puncture, “move-the-cap” techniques, and retrograde crossing) for CTOs with ambiguous proximal caps. The Asia Pacific CTO Club (APCTO) algorithm recommends IVUS first, followed by retrograde crossing if IVUS cannot clarify the anatomy, and, if there are proximal caps with a favorable side branch, then IVUS-guided proximal cap puncture may be preferred (8). In our case, the D1 vessel within the CTO body was successfully crossed by the antegrade wire. However, following the “move-the-cap” technique with a knuckled wire resulted in the perforation complication. Therefore, if we had used IVUS to guide the CTO entry point puncture, we may have avoided such a complication. In this scenario, the antegrade approach to recanalize the LAD CTO was hindered. In cases of hemodynamic instability, the immediate implantation of covered stents from the D1 vessel to the proximal LAD could be an option, but as a result, LAD CTO recanalization would become more challenging in the future. Alternatively, emergency coronary artery bypass grafting (eCABG) surgery, as a last resort, could effectively revascularize the LAD lesions and the D1 vessel. In a retrospective analysis of coronary perforations treated with covered stents in the United States, eCABG was required in 6.3% (67 out of 1,069) of cases. In addition, in-hospital mortality occurred in 12.4% (134 out of 1,084) of perforation cases (9). In our case, the hemodynamics remained stable after tamponade balloon use, so the operator was able to perform DARTs to recanalize the LAD CTO lesion and intentionally create efficient dissection flaps. The concept of this technology was that the retrograde wire in the subintimal space was intentionally manipulated away from the perforation guidewire plane, ensuring that the two wires were not in close proximity. The aim was to generate sufficient subintimal tissue. After the retrograde approach succeeded, balloon inflation crushed the body of the lesion and subintimal flaps, shifting them to the side of the perforation and sealing it with DES deployment. Our case and previous cases (5–7) indicate that the benefit of this technique obviates the need for coiling or covered stents. In addition, drug-eluting stent implantation likely provided the advantage of lower restenosis rates. If the perforation had not been sealed by this innovative technique, the subsequent deployment of covered stents would have been necessary. This innovative approach is an alternative to covered stents and can be intentionally deployed by an experienced operator as a bailout solution. However, there are limitations to this technique. The operator must be highly proficient in the technique transition according to the CTO hybrid algorithm, and maintaining hemodynamic homeostasis is critically important during this procedure. If there are insufficient subintimal flaps, the high balloon pressure during pre- or post-dilation would exacerbate vessel wall injury. This technique should only be utilized by experienced operators when the anticipated benefits clearly outweigh the potential risks.

Van Chien et al. (10) recently performed a novel technique using an injection of combined Histoacryl (n-Butyl-2-Cyanoacrylate) and Lipiodol (ethiodized oil) to seal coronary perforations, providing a potentially safer and more effective method compared to the traditional CSs. As noted in previous reports (3, 4), in this case, the side branch became reoccluded due to the displacement of the dissection flaps. To avoid the potential risk of tearing the dissection flaps during balloon dilation at the bifurcation, we opted not to rewire the D1 vessel.

## 4 Conclusion

This innovative approach to managing coronary artery perforation using autologous flaps in CTO-PCI serves as an effective alternative to covered stents. However, surgeons should pay attention to the potential risks and ensure hemodynamic stability throughout the procedure.

## Data Availability

The original contributions presented in the study are included in the article/Supplementary Material, further inquiries can be directed to the corresponding author.
